# So Few COVID-19 Cases in Taiwan: Has Population Immune Health Played a Role?

**DOI:** 10.3389/fpubh.2021.676750

**Published:** 2021-06-14

**Authors:** Wen-Ta Chiu, Jeremiah Scholl, Yu-Chuan Jack Li, Jonathan Wu

**Affiliations:** ^1^AHMC Health System, Alhambra, CA, United States; ^2^AESOP Technology, San Francisco, CA, United States; ^3^College of Medical Science and Technology, Graduate Institute of Biomedical Informatics, Taipei Medical University, Taipei, Taiwan; ^4^International Center for Health Information Technology (ICHIT), College of Medical Science and Technology, Taipei Medical University, Taipei, Taiwan; ^5^Research Center of Big Data and Meta-Analysis, Wan Fang Hospital, Taipei Medical University, Taipei, Taiwan; ^6^Department of Dermatology, Wan Fang Hospital, Taipei Medical University, Taipei, Taiwan

**Keywords:** COVID-19, public health, heath promotion, Taiwan, pandemic

## Introduction

**Figure 1:**
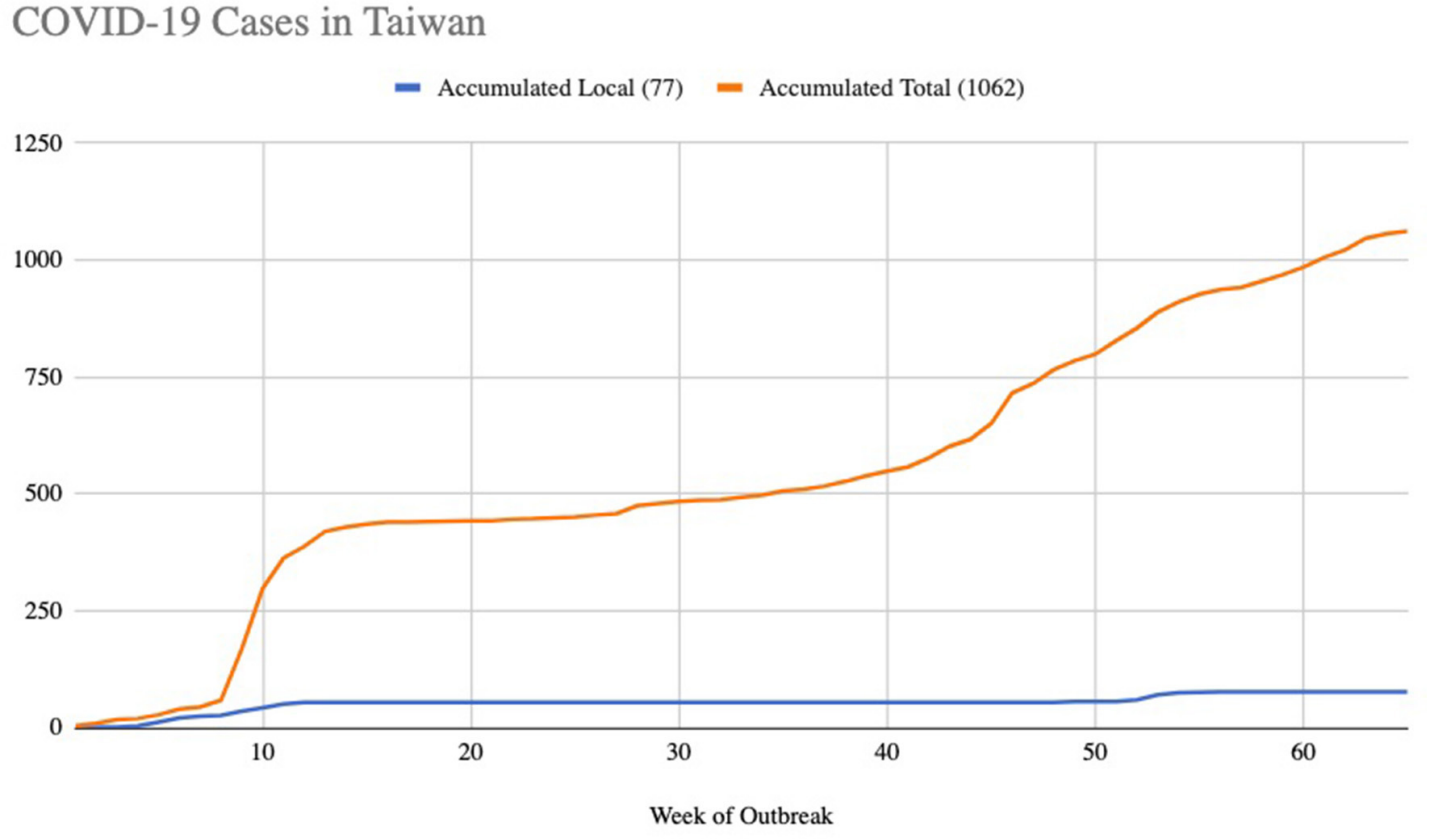
Accumulated number of COVID-19 cases in Taiwan.

Taiwan and China have very close ties, with more than 400,000 Taiwanese citizens working there. It was thus expected that Taiwan would be one of the hardest hit countries in the world by COVID-19 when the outbreak started. However, Taiwan has had extra ordinary success in controlling and managing the pandemic. By April 12, 2021 for example, Taiwan had only 1,058 confirmed COVID-19 cases and seven deaths, one of the lowest in the world. At one point they managed to go over 250 days without a confirmed case of local transmission, and local cases accounted for 7.2% (Figure 1).

This has lead to general interest in how Taiwan managed to contain COVID-19, and several papers have been published that describe the Taiwanese public health response and other success factors (1, 2). The measures they described include effective response systems previously in place due to the 2003 SARS experience, early proactive measures, contact tracing, promoting transparency, public education, enforcing social cohesion, and fostering a public sense of urgency. These actions were strengthened by a central command center that managed the response, and a public that has high trust in public health authorities.

These containment strategies are clearly the main cause of successful control of COVID-19 in Taiwan. The level of mobilization and fast reaction by the health authorities and the public saved many lives and protected the country from economic damage. Interestingly however, evidence has also emerged that a few more people may have contracted COVID-19 than reported in the official case numbers, but the vast majority of cases were asymptomatic and did not lead to an uncontrolled outbreak like experienced in other countries. It is thus interesting to discuss if population immune health may have provided an additional layer of protection from the outbreak, and to discuss policy measures Taiwan has had in place that may have helped deliver this immunity to it's population.

## Possible SILENT and Asymptomatic COVID-19 Cases in Taiwan

The hypothesis that more people in Taiwan contracted COVID-19 than reported in the official case numbers is supported by two key data points.

The first data point is an outbreak on a military warship, with 36 crew members infected in April 2020 after returning from a diplomatic visit to Pacific Island countries (3). Many people had already left the warship, and spent 3 days in the community visiting 90 sites by the time the outbreak was identified. This resulted in the recall and quarantine of 744 people. However, all cases were asymptomatic or mild and only one case had short hospitalization without any deaths. There also were no new cases and no community spread after May 5th, 2020.

The second data point is a study by Veteran General Hospital, Taipei. They conducted serological antibody tests on 14,765 people, and found a positive rate for COVID-19 of 5/10,000 persons. This data lead the authors to estimate that ~11,800 people in Taiwan had come in contact with COVID-19 (4).

These two data points do suggest that COVID-19 had a more significant opportunity to rise to a level of uncontained community spread than you would expect given so few cases that reached hospitals. It is thus interesting to ask, “How did the population handle contracting COVID-19 so well in Taiwan?” We hypothesize that this was helped by two factors related to positive immune health of Taiwan's population, both aided by Public Health and Health Promotion policies. These two factors are cross-immunity and general health from nutrition and exercise.

## Public Health Policy and Cross-Immunity

One issue that Taiwan may have benefited from is cross immunity provided by effective vaccine programs and previous infections. With respect to vaccines, there is evidence that they can induce “trained” non-specific innate immune cells into functioning more effectively against broader infections than just the one being vaccinated against. In this respect, vaccinating against other infections may have helped the Taiwan population obtain some immunity from COVID-19.

This hypothesis is supported by several studies showing protection from COVID-19 by vaccines for other infections. Epidemiological analyses for example, has shown BCG vaccination for Tuberculosis to be associated with reduced COVID-19 cases and mortality, and there are seven ongoing clinical trials testing it (5, 6). The epidemiological analysis is somewhat controversial though, as one multi country epidemiological study showed no association between BCG vaccination policy and COVD-19 spread rate (7). The hypothesis is supported however by cohort analysis of Health Care Workers in Los Angeles County, USA. This analysis showed that previous BCG vaccination was correlated with lower self-reported COVD-19 symptoms, and lower seroprevelance of COVID-19 specific antibodies (8).

There is also evidence of protection from other vaccines. A study from the USA for example has shown positive results for antibodies from the MMR vaccine given for measles, mumps, and rubella in COVID-19 outcomes (9). A study from Italy also provides evidence that Influenza vaccine provided some protection from COVID-19 deaths among the elderly (10). A study from Adana, Turkey also showed that circulating antibodies from a variety of vaccines, including MMR and Influenza, is correlated with better COVID outcomes (11).

Taiwan has higher vaccination rates compared to most other countries, due to its National Health Insurance system that covers 99.8% of the population. This suggests that it may have had an edge in cross-immunity entering the pandemic. The MMR vaccination rate is over 98% for the past 11 years for example, and the BCG vaccination rate is over 98% during the same period. Taiwan also has high vaccination rates for influenza, polio and other conditions. Together all of this may have provided a measure against COVID-19 for the Taiwanese population, reducing the odds that a person coming in contact with the virus would end up becoming contagious and/or would eventually infect someone else.

It should be noted that in addition to cross-immunity protection from vaccines, the Taiwan population might have above average natural cross-immunity from previous coronavirus outbreaks. It has been suggested for example that exposure to SARS and more common coronaviruses might explain why Asia in general had better outcomes than other parts of the world (12, 13). While it is unclear to what extent the Taiwan population has had greater exposure to common cold coronaviruses, high population density suggests this is likely. Taiwan was also one of the hardest hit countries by the 2003 SARS outbreak. Taiwan had 668 cases and 281 deaths, the 2nd highest death rate in the world, and a total of 151,270 persons were quarantined before SARS disappeared in July, 2003.

Since Taiwan was one of the worst hit countries by SARS, it is worth discussing why Taiwan might have had a much better relative outcome in trying to control COVID-19. It does not seem likely that changes in population immunity between the two outbreaks explains the difference. More likely is that the SARS-CoV outbreak was extremely limited globally in comparison to COVID-19. The primary determinant of impact on a country with SARS-CoV was proximity to the origin of the outbreak, and frequency of travelers from that region. It is thus difficult to draw any conclusions from SARS-CoV with respect to population immunity by looking at relative impact in different countries.

## Health Promotion Policy With Nutrition and Exercise

Beyond vaccination a second factor that may be provided significant immune protection in Taiwan is health promotion measures focusing on nutrition and exercise. Health Promotion is taken very seriously in Taiwan, with 166 cities and 45 non-governmental organizations having joined the Alliance for Healthy Cities (AFHC) supported by the WHO. This, and other measures, have helped Taiwan to maintain a low obesity rate (7.2%) as well as providing other benefits (14).

Early on in the pandemic it was shown that low obesity rate in a population had an even stronger correlation with success in controlling the outbreak than rapid border closures, and wide-spread testing (15). The World Obesity Federation has also reported that countries with more than half the population classified as overweight had COVID-19 death rates 10 times higher than those that did not (16). The World Obesity Foundation thus recommend that governments invest in obesity prevention to help mitigate the impact of future pandemics.

Obese people are more likely to have serious complications, and a higher viral load, and for influenza viruses it has also been shown that they shed the virus for a longer period of time. High levels of obesity will lead to more patients with symptoms, and at hospitals that can spread the infection. It is thus likely that obesity in the population not only put the population of obese people at higher risk, but also makes pandemic management in general more difficult for everyone. Beyond protection from obesity, physical activity in general has also shown to have protective benefits against Covid (17).

Micronutrients have also been discussed as a contributor to immune health that likely had an impact on COVID-19 infections (18). Deficiencies in Vitamins D, C, and Zinc for example are implicated for vulnerability to infections in general, and also specifically for COVID-19 (19–21). There appears to be much higher variability in COVID-19 outcomes related to micronutrient profile than with vaccinations and obesity however, and the strong country level data that exists for vaccination rates and obesity rates does not been published with respect to differences in country level profiles for micronutrients. So, population level micronutrient profiles may not provide as much protection as cross-immunity and low obesity rates.

Taiwan was still in a strong position with respect to micronutrient profile of the population however. For example, Taiwan has a generally strong nutrition profile for Vitamins D and Zinc among the elderly (22, 23). In the USA 61% of elderly are estimated to be Vitamin D deficient. In Taiwan however only 27.5% of those 60–69, 16.8% of 70–79 years, and 15.7% of those older than 79 are estimated to be deficient. Levels of zinc deficiency among the elderly are also measured to be very low in Taiwan, but in the US 20–25% of older adults are thought to not get enough zinc. For Vitamin C the average person in Taiwan consumes around 142 mg per day compared to only 102 mg for men in the US and 83 mg for women in the US.

Together this vitamin and mineral profile could have provided extra protection for the highest risk age group, making the outbreak easier to manage.

## Discussion

We must re-emphasize that the primary reason effective COVID-19 pandemic control occurred in Taiwan was because of quick and diligent reactions by public health authorities since the beginning of the outbreak. This was aided by previous lessons Taiwan learned during the SARS outbreak in 2003, and supported by an overall strong relationship between public health authorities and the public that has been maintained through offering National Health Insurance. The strong trust between public health authorities and the public was of course critical to ensuring adherence to COVID-19 safety measures (24).

But also, in this paper we have presented analysis suggesting that the pandemic might have been more manageable from the start due to population immune health. This immune health was aided by a long term focus on population health through immunization by the National Health Insurance system, from health promotion policies, and also possibly from naturally obtained cross-immunity.

The Taiwan experience adds to evidence suggesting that general population health is an important part of pandemic planning, and that authorities should consider improved pandemic resistance as a likely benefit of population health investments. Some of this evidence however remains stronger than others, so further research is still needed to clarify which investments are likely to have highest impact. In particular, while the evidence of benefits from low obesity rates and healthy levels of exercise in preventing infections are quite strong, further research still needs to be conducted to confirm the connection between common vaccinations and general coronavirus immunity. Ongoing clinical trials and further population level analysis should shed further light on the issue, and clarify the role that these vaccinations might play in pandemic preparedness for the future.

## Author Contributions

W-TC conceived the paper, wrote an initial draft, and prepared the data about COVID-19 in Taiwan. JS helped identify additional important references, to interpret the results in context of those references, and was primary author from initial draft until the submitted version. JW and Y-CL provided editorial over site and strategic thoughts on interpreting the data and studies referenced. All authors contributed to the article and approved the submitted version.

## Conflict of Interest

The authors declare that the research was conducted in the absence of any commercial or financial relationships that could be construed as a potential conflict of interest.
